# Update on the Mapping of Prevalence and Intensity of Infection for Soil-Transmitted Helminth Infections in Latin America and the Caribbean: A Call for Action

**DOI:** 10.1371/journal.pntd.0002419

**Published:** 2013-09-19

**Authors:** Martha Idalí Saboyá, Laura Catalá, Rubén Santiago Nicholls, Steven Kenyon Ault

**Affiliations:** Pan American Health Organization/World Health Organization, Washington, District of Columbia, United States of America; London School of Hygiene and Tropical Medicine, United Kingdom

## Abstract

It is estimated that in Latin America and the Caribbean (LAC) at least 13.9 million preschool age and 35.4 million school age children are at risk of infections by soil-transmitted helminths (STH): *Ascaris lumbricoides*, *Trichuris trichiura* and hookworms (*Necator americanus* and *Ancylostoma duodenale*). Although infections caused by this group of parasites are associated with chronic deleterious effects on nutrition and growth, iron and vitamin A status and cognitive development in children, few countries in the LAC Region have implemented nationwide surveys on prevalence and intensity of infection. The aim of this study was to identify gaps on the mapping of prevalence and intensity of STH infections based on data published between 2000 and 2010 in LAC, and to call for including mapping as part of action plans against these infections. A total of 335 published data points for STH prevalence were found for 18 countries (11.9% data points for preschool age children, 56.7% for school age children and 31.3% for children from 1 to 14 years of age). We found that 62.7% of data points showed prevalence levels above 20%. Data on the intensity of infection were found for seven countries. The analysis also highlights that there is still an important lack of data on prevalence and intensity of infection to determine the burden of disease based on epidemiological surveys, particularly among preschool age children. This situation is a challenge for LAC given that adequate planning of interventions such as deworming requires information on prevalence to determine the frequency of needed anthelmintic drug administration and to conduct monitoring and evaluation of progress in drug coverage.

## Author Summary

Soil-transmitted helminths (STH) are part of the group of neglected infectious diseases (NID) in Latin America and the Caribbean (LAC), and are associated with several adverse chronic effects on child health. Although control interventions such as periodic administration of anthelmintic drugs, health education, improved access to safe water and sanitation, among others, are acknowledged to be an important means to reduce morbidity and to achieve control, epidemiological information on prevalence status is lacking at the lowest sub-national administrative levels (municipalities, districts or provinces) in many countries thus hindering decision making regarding not only the treatment, but also the monitoring of progress in deworming coverage, the assessment of epidemiological impact on parasite prevalence and load and, therefore, the achievement of the overall public health goals. Epidemiological surveys can be expensive and require time and effort for their implementation, which could explain the low number of studies published with data on prevalence and intensity of infection in the Americas. The use of alternative methodologies, for instance those based on geographical information systems and remote sensing technologies, or of sentinel surveillance in schools may help countries in the task of collecting information and support the implementation of integrated control programs against STH.

## Introduction

Helminth infections impose a great and often silent burden of morbidity and mortality on poor populations in developing countries. The most common helminth infections are caused by soil-transmitted helminths (STH): roundworms (*Ascaris lumbricoides*), whipworms (*Trichuris trichiura*), and hookworms (*Necator americanus* and *Ancylostoma duodenale*). Worldwide estimates suggest that *A. lumbricoides* infects 1.221 billion people, *T. trichiura*, 795 million, and hookworms, 740 million. Infections occur most frequently in the Americas, China and East Asia, and Sub-Saharan Africa [1].

STH are amongst the most prevalent pathogenic organisms on the planet, estimated to infect almost one-sixth of the global population with the highest rates among school-age children (SAC) who are frequently infected with two or more species at a time [2]. Stunting usually occurs between 6 months and 2 years of age, overlapping with the period in which STH begin to emerge [3]. STH infection primarily affects physical and cognitive development [4]. *A. lumbricoides* can cause malnutrition; hookworms damage the intestinal mucosa leading to bleeding, loss of iron and anemia, and infections by *T. trichiura* cause chronic reduction of food intake [5]. During pregnancy, mild or severe infections by hookworms can cause anemia in the mother and damage to the fetus, leading to low birth weight [6]. In areas where helminths are common, deworming activities can be done once or twice a year among the population at risk (those with no access to improved sanitation facilities), including deworming for pregnant women after the first trimester. Deworming during pregnancy reduces severe maternal anemia, increases birth weight and reduces infant mortality [7]. Thus, regular treatment against helminth infections produces both immediate and long-term benefits, contributing significantly to improving the growth and cognitive development of affected individuals, especially children.

In 2001, the World Health Assembly adopted Resolution WHA/54.19 [8] urging all Member States where STH are endemic to attain “a minimum target of regular administration of chemotherapy to at least 75% and up to 100% of all SAC at risk of morbidity by 2010”. On October 2009, the Directing Council of the Pan American Health Organization (PAHO) approved Resolution CD49.R19 [9] stating the commitment of PAHO's Member States to eliminate or reduce neglected diseases, among them STH, to levels such that they are no longer considered public health problems by the year 2015, and hence help to achieve the Millennium Development Goals. In PAHO's Resolution, STH and schistosomiasis were classified as diseases whose prevalence can be drastically reduced with available cost-effective interventions. Regarding STH, the following goal to be reached by 2015 was defined: reducing prevalence among SAC in high risk areas (prevalence >50%) to less than 20% as measured by quantitative egg count in feces. The Resolution also mentioned several interventions to reach the STH control goal, especially those related with improved access to safe water and basic sanitation, preventive chemotherapy and health education through inter-sectoral collaboration.

According to WHO/PAHO estimates, in LAC there were 13.9 million preschool-age children (PSAC) and 35.4 million SAC in need of preventive chemotherapy for STH in 2012. These estimates have been calculated based mainly on the percentage of people without access to improved sanitation facilities, differentiated by rural and urban areas, due to the fact that STH prevalence and intensity of infection in LAC are not well mapped [10].

The purpose of this paper is to present the status of the mapping of prevalence and intensity of STH infection and to identify information gaps in LAC for the 2000–2010 period based on a literature search. This study is a call for action in LAC to address the existing information gaps in order to prioritize integrated interventions for STH control based on solid evidence, increase efforts towards reduction of the morbidity caused by these parasites, and reach the targets in the WHO and PAHO resolutions.

## Materials and Methods

### Data selection

A wide literature search was conducted to collect data on STH prevalence and intensity of infection (*Ascaris lumbricoides*, *Trichuris trichiura* and hookworms) among preschool (1–4 years of age) and school-age (5–14 years of age) children for the 2000–2010 period at the lowest subnational administrative levels in LAC countries (Checklist S1). The decision to include studies published between 2000 and 2010 was taken arbitrarily by the authors considering this 10-year period to have sufficient updated information on the status of STH mapping in the Region to establish the current information availability, reflecting the level of interest on this issue in the Region. A database was built including information from 236 studies, of which 120 met the inclusion criteria [11]–[130]. There are 45 countries and territories in LAC with at least 13,591 units at second subnational level that may be districts, municipalities or provinces depending on the geopolitical structure of each country or territory. Once the information was collected, a preliminary report was published in PAHO's website [131]. That document is the main information source for the analysis presented here.

The scientific literature search was done through the online databases of PubMed (including MEDLINE), LILACS (including SciELO), BIREME and Cochrane. Additionally, we carried out a search of information published in the websites of health ministries, NGOs and FBOs reporting data on deworming activities to PAHO and WHO from 2005 to 2010, as well as information published on the websites of PAHO country offices in LAC. The online database search was done using MeSH terms to facilitate an ample retrieval of published information on STH prevalence and intensity of infection in LAC. The following MeSH terms and subheadings were used for searches on PubMed: ((“Helminthiasis”[Mesh] OR (“Helminthiasis/epidemiology”[Mesh] OR “Helminthiasis/parasitology”[Mesh] OR “Helminthiasis/statistics and numerical data”[Mesh]))) AND (“Child”[Mesh] OR (“Child/epidemiology”[Mesh] OR “Child/statistics and numerical data”[Mesh])). The following terms were also used for searches on PubMed to recover more papers: 1) ‘Prevalence intestinal parasites child’ restricted by country, sub-regions in LAC (Central American Isthmus, Latin Caribbean, Andean area, Southern Cone, Non-Latin Caribbean) and publication year of study; 2) ‘Soil transmitted helminths prevalence’ restricted by country, sub-regions in LAC (Central American Isthmus, Latin Caribbean, Andean area, Southern Cone, Non-Latin Caribbean) and publication year of study; and 3) ‘*Ascaris lumbricoides* or *Trichuris trichiura* or hookworms or *Necator americanus* or *Ancylostoma duodenale*’ by country, sub-regions in LAC (Central American Isthmus, Latin Caribbean, Andean area, Southern Cone, Non-Latin Caribbean) and publication year of study. Additionally, for the search of papers in BIREME and LILACS databases, the following terms were used: 1) “Helminthiasis” AND “prevalence” by country and LAC; and 2) “Intestinal parasites” AND “prevalence” by country and LAC.

The following were the inclusion criteria: 1) studies with data on prevalence and intensity of infection published from 2000 to 2010 including the geographical location of the study so as to enable the identification of the municipal or local level; and 2) studies including STH prevalence data disaggregated by age groups. The following were the exclusion criteria: 1) studies undertaken before 1995; 2) reports with data on intensity of infection not using WHO classification parameters (mild, moderate and high), and 3) studies with no data on their geographical location or including data on prevalence and intensity of infection of limited use for the present analysis (e.g. studies reporting prevalence data only for adults, or studies in children with eosinophilia). Given the limited number of data published on STH prevalence and intensity of infection in the Region, no restriction was established regarding sample size, type of study (community-based or among school children, base line or intervention monitoring) or laboratory diagnostic methods used, as the aim was not to verify the existence of accurate data for the two indicators in the Region, but to explore existing information and gaps on mapping (Figure S1).

After reviewing the articles and reports, information was extracted from those that met the inclusion criteria and then a database was built with the following variables: name of country; name of geographical location of study; name of geographical locations within the first and second subnational levels corresponding to the geographical location of the study (in those cases where no information on this regard appeared, the publication year was recorded), sample size, STH prevalence, prevalence of infection by species, intensity of infection and age group of study subjects. Each value of prevalence and intensity of infection registered on the database was denominated a data point. In those studies that included data for several geographical locations at the lowest subnational level (e.g., municipalities), data were registered for each of these locals and, therefore, we were able to extract more than one prevalence or intensity of infection data point from several studies.

### Analysis

A descriptive analysis of the number of studies including data on prevalence and intensity of infection published from 2000 to 2010 was done by country, age group and prevalence and intensity of infection range. Although the search was restricted to studies published from 2000 to 2010, some authors included results from surveys conducted before the study publication date, and for this reason our analysis included data only from studies carried out from 1995 onwards. Besides the analysis of frequency and proportion distributions, the geographic locations of prevalence and intensity of infection data points for preschool and school age children were mapped, as this was useful to visualize gaps in data publishing. The database was made with MS Excel 2010 and the analysis with Tableau 7.0.

## Results

A total of 236 publications were found, of which 120 met the selection criteria established for the study; the publications corresponded to 18 countries: Brazil (39 publications, 32.5%), Argentina (11, 9.2%), Colombia (10, 8.3%), Venezuela, Mexico, Ecuador (9, 7.5% each), Peru (7, 5.8%), Cuba (5, 4.2%), Honduras, Bolivia (4, 3.3% each), Guatemala (3, 2.5%), Haiti, Costa Rica, Belize (2, 1.7% in each country), Saint Lucia, Paraguay, Nicaragua and Guyana (1, 0.8% each). All publications were articles published in scientific journals. Although some documents were found in the websites of health ministries, NGOs and FBOs, none of them included information meeting the inclusion criteria. The studies recovered by the Cochrane database had no information on their geographic location and, therefore, they were not considered.

A total of 335 data points on STH prevalence were registered and analyzed for 18 countries out of which 12.0% were for preschool-age children (40 prevalence data points), 56.7% for school-age children (190 prevalence data points) and the remaining 31.3% (105 prevalence data points) were registered for children under 15 years of age because authors did not differentiate by age groups, although they did state in the methodology that the study had been undertaken among children under 15 years of age. Another 687 data points on prevalence by STH species were also extracted, of which 40.2% corresponded to *A. lumbricoides*, 35.5% to *T. trichiura*, 19.5% to hookworms (undifferentiated by species), 2.9% to *A. duodenale*, and 1.9% to *N. americanus*. Additionally, 151 data points were extracted for intensity of infection.

**Figure 1:**
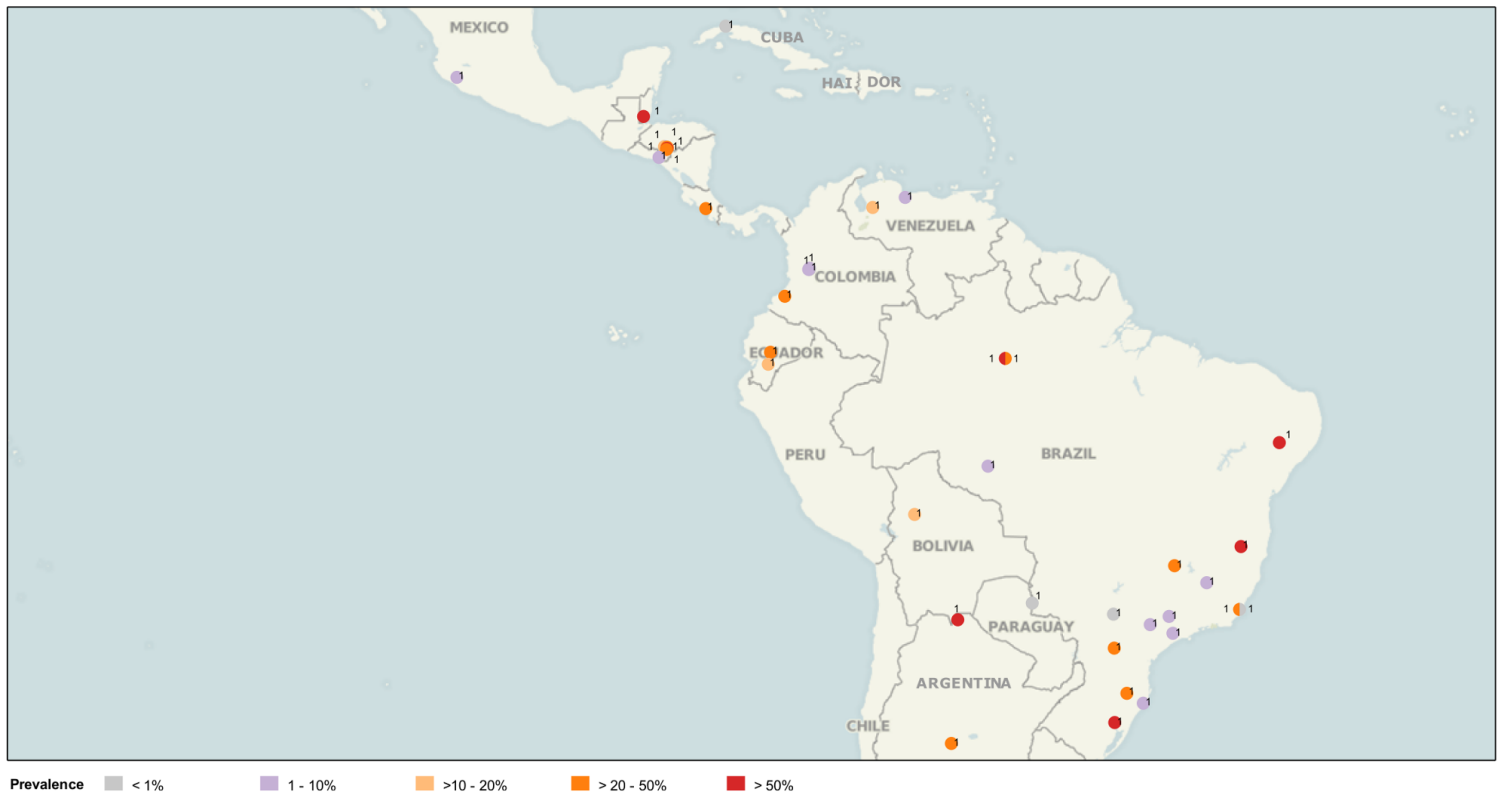
Data points found for STH prevalence among PSAC in LAC, 2000–2010. *Source*: PAHO based on literature search for Latin American and the Caribbean. Numbers indicate number of data points found for the same geographical location. (Image created using www.tableausoftware.com/mapdata).

**Figure 2:**
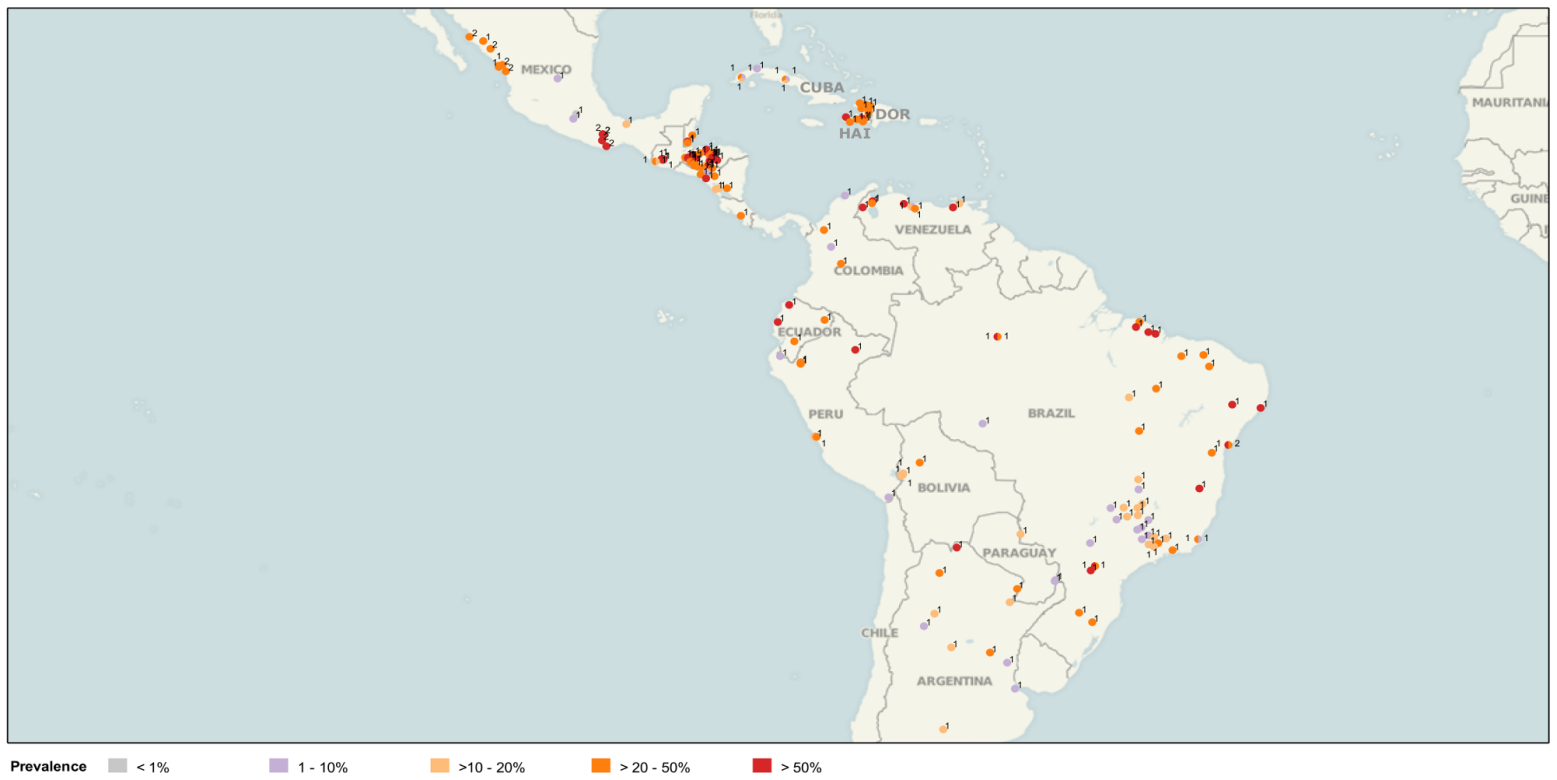
Data published for STH prevalence among SAC in LAC, 2000–2010. *Source*: PAHO based on literature search for Latin American and the Caribbean. Numbers indicate number of data points found for the same geographical location. (Image created using www.tableausoftware.com/mapdata).

The main characteristics of the 335 data points for STH infection prevalence were the following: 1) 27.8% of prevalence data points showed values over 50%, 34.9% between >20 and 50%, and 37.3% below 20%; 2) 63% of prevalence data points corresponded to data published by four countries (Argentina, Brazil, Honduras and Mexico); 3) 35% or more of STH prevalence data from Belize, Ecuador, Guatemala, Honduras, Mexico and Venezuela showed values over 50%. Prevalence ranges found in each country are shown in Table 1. The geographic locations of STH prevalence data points for PSAC and SAC by prevalence ranges are shown in Figures 1 and 2.

**Table 1:** Number and relative frequency of data points found for STH prevalence ranges by country, LAC 2000–2010.

Country	<1 (%)	1–10 (%)	>10–20 (%)	>20–50 (%)	>50 (%)	Data source
Argentina	1 (3.1)	6 (18.8)	7 (21.9)	7 (21.9)	11 (34.4)	[11]–[21]
Belize	-	-	-	2 (40)	3 (60)	[22], [23]
Bolivia	-	3 (25)	6 (50)	2 (16.6)	1 (8.3)	[24]–[27]
Brazil	5 (5.9)	21 (23.9)	18 (20.5)	25 (28.4)	19 (21.6)	[20]–[66]
Colombia	1 (9)	7 (63.6)	-	3 (27.3)	-	[67]–[76]
Costa Rica	-	-	-	4 (100)	-	[77], [78]
Cuba	1 (11.1)	3 (33.3)	2 (22.2)	3 (33.3)	-	[79]–[83]
Ecuador	-	1 (9)	1 (9)	4 (36.4)	5 (45.5)	[84]–[92]
Guatemala	-	-	1 (7.1)	8 (57)	5 (35.7)	[93]–[95]
Guyana	-	-	-	1 (100)	-	[96]
Haiti	-	-	-	10 (83.3)	2 (16.7)	[97]–[98]
Honduras	-	3 (4.5)	8 (12.1)	26 (39.4)	29 (43.9)	[99]–[102]
Mexico	1 (3.6)	5 (17.9)	1 (3.6)	11 (39.3)	10 (35.7)	[103]–[111]
Nicaragua	-	-	2 (40)	2 (40)	1 (20)	[112]
Paraguay	-	2 (100)	-	-	-	[113]
Peru	-	3 (27.3)	2 (18.2)	5 (45.4)	1 (9)	[114]–[120]
Saint Lucia	-	3 (37.5)	4 (50)	1 (12.5)	-	[121]
Venezuela	-	4 (25)	3 (18.8)	3 (18.8)	6 (37.5)	[122]–[130]

- No data points found.

A total of 151 infection intensity data points from seven countries (Argentina, Bolivia, Brazil, Colombia, Ecuador, Honduras and Venezuela) were registered and analyzed. The following were their main characteristics: 1) The largest number of data points was found for Honduras (41.7%), Bolivia and Brazil (15.9% each); 2) 56.9% of infection intensity data corresponded to SAC, 17.8% to PSAC and 25.1% to children under 15 years of age; 3) 37.1% showed light, 34.4%, moderate and 28.5% heavy intensity infections (Table 2); 4) 65.1% of the data points on heavy intensity infections corresponded to *A. lumbricoides* (28 data points), and 5) no data on intensity of infection published after 2005 were found.

**Table 2:** Number and relative frequency of data points found for each category of STH intensity of infection by country, 2000–2010.

Country	Light (%)	Moderate (%)	Heavy (%)	Data Source
Argentina	0 (0)	0 (0)	2 (4.7)	[14]
Bolivia	8 (14.3)	8 (15.4)	8 (18.6)	[24]
Brazil	9 (16.1)	8 (15.4)	7 (16.3)	[53], [63]
Colombia	3 (5.4)	3 (5.8)	3 (7)	[67]
Ecuador	2 (3.6)	2 (3.9)	5 (11.6)	[85], [89]
Honduras	27 (48.2)	24 (46.2)	12 (27.9)	[99], [100]
Venezuela	7 (12.5)	7 (13.5)	6 (13.9)	[123], [126]
**Total data points found by level of intensity**	56 (100%)	52 (100%)	43 (100%)	

The most recent year in which prevalence data points were found varied among countries. Regarding prevalence data points for SAC, and taking as the most recent data those from studies conducted after 2005, we observed that 10 countries (out of 16 with data points) had studies conducted from 2005 to 2010. As for preschool age children, six countries (out of 11 with data points) had studies, while nine countries had studies for children under 15 years of age (out of 16 with data points).

## Discussion

A total of 335 data points on STH prevalence from 18 LAC countries were found in the 120 articles included in the present study that were then registered and analyzed; 12.0% of the data were for PSAC, 56.7% for SAC and 31.3% for children under 15 years of age. We found that 60% of the prevalence data points showed prevalence levels above 20%. If these data were to be used for decision making regarding deworming in those geographic areas, it could be concluded that in more than half of such places deworming would be necessary for all children under 15 years of age (excluding children under 1 year of age) at least once a year. We also registered and analyzed 151 infection intensity data points from seven countries, of which 37.1% showed light, 34.4%, moderate and 28.5%, heavy intensity infections; no data published on intensity of infections were found after 2005. Our analysis suggests that there is a significant gap in data published on STH prevalence and intensity of infection in LAC, especially for PSAC.

No restriction was established regarding sample size, sample randomization, type of study (community-based or among SAC, baseline or post-intervention monitoring) or laboratory diagnostic methods used, and, therefore, the study does not allow for making any inferences. Nevertheless, this information is useful to recognize existing data gaps on prevalence and intensity of infection for STH, independently of the quality of the few data found, and emphasizes the need to make a call for action aimed at collecting evidence-based information required to define needed deworming activities for public health purposes in LAC countries. The results also emphasize the need to promote the development of studies that are epidemiologically robust, with a certain standardization of both the epidemiological and laboratory methods, following WHO guidelines, that will provide reliable information on the epidemiological situation of STH and allow for comparability between studies done in different geographical regions and at different points in time. Another limitation of the study could be the fact that the data from some reports published by health ministries and organizations (NGOs and FBOs), as well as from undergraduate and graduate theses and other type of documents containing information on studies about prevalence and intensity of infection could have been omitted due to the methodology used for data recovery that focused mainly on published indexed data accessible on the web. Due to the aforementioned limitations and the bias that this could represent for this study, inferences or use of these data to estimate prevalence and intensity of infection for STH and its distribution in LAC, or for making decisions on deworming activities to be implemented in any area of the 18 countries included in the analysis should not be done. Countries are encouraged to conduct their own scientifically sound studies of prevalence and intensity of STH infection, where data gaps are seen.

During the period from January 2000 to June 2010 data on STH prevalence and intensity of infection at the local level were recovered only for 18 LAC countries (Argentina, Belize, Bolivia, Brazil, Colombia, Costa Rica, Cuba, Ecuador, Guatemala, Guyana, Haiti, Honduras, Mexico, Nicaragua, Paraguay, Peru, Saint Lucia and Venezuela) despite the fact that it is estimated that at least 30 countries in the Americas are endemic for STH infections according to a document published by PAHO in 2010 [131]. In this report, LAC countries were classified into four groups based on the estimated number of PSAC and SAC at risk of STH infections, as well as on the presence of other neglected infectious diseases (NIDs) targeted for preventive chemotherapy as one of the tools to reach their control and elimination. This classification of countries in four groups was done from analysis of quantitative and qualitative variables. The group of quantitative variables included sanitation coverage, population at risk for STH, deworming coverage on PSAC and SAC population, population at risk, prevalence and treatment coverage for onchocerciasis, schistosomiasis, blinding trachoma and lymphatic filariasis. The qualitative variables included inter-programmatic actions already in implementation in the countries, partners supporting deworming, needs of technical cooperation, progress on mapping, and opportunities for integrated actions.

These four groups included 33 countries as follows: the first group comprised 11 countries (Bolivia, Brazil, Ecuador, Guatemala, Guyana, Haiti, Mexico, Peru, Dominican Republic, Saint Lucia and Suriname) concentrating 66.8% of PSAC and 67.4% of SAC at risk of STH infections in the Region. The second group included six countries (Belize, Colombia, El Salvador, Honduras, Panama and Venezuela) with 26.8% of PSAC and 26.1% of SAC at risk. The third group comprised three countries (Argentina, Paraguay and Nicaragua) with 5.4% of children at risk from both age groups, and the fourth group of 13 countries registering 1% of children at risk from both age groups. If deworming activities were focused in the 17 countries in groups 1 and 2, 94% of PSAC and SAC at risk of STH infections in the Region would be reached and protected [132].

Of the 335 data points from the 18 LAC countries analyzed in the study, 62.7% of data points showed STH infection prevalence above 20% (including both ranges, 20–50% and >50%). If these data were representative of the geographic areas to which they correspond, and if a country was interested in implementing deworming using these data without waiting for data of a survey of prevalence to make a decision on number of treatment rounds needed, it would be necessary to conduct deworming at least once a year for all children aged between 1 and 15 years there. According to information provided by PAHO's Regional NID Program, by 2011 a total of eight countries (Dominican Republic, Guyana, Haiti, Mexico, Belize, Honduras, Nicaragua and Cuba) had deworming programs at national and subnational levels while four other countries had started updating their mapping of prevalence and intensity of infection at national level (Brazil, Ecuador, El Salvador and Honduras), while Suriname had finished a national survey in 2010. As a result of the advocacy activities waged by PAHO and other regional partners, and based on meetings held with national health authorities, it is expected that by 2013 at least 11 countries from groups 1, 2 and 3 prioritized by PAHO (Bolivia, Dominican Republic, Guatemala, Guyana, Haiti, Peru, Colombia, Panama, Argentina, Paraguay and Venezuela) will update their mapping of STH prevalence and intensity of infection, and that four countries will conduct mapping to evaluate the impact of their national deworming programs (Belize, Mexico, Nicaragua and Saint Lucia) (Table 3).

**Table 3:** Overview of current status of implementation of deworming programs and progress on STH mapping, 2011–2012.

PAHO's priority group	Country	Deworming activities for STH up to 2011	Mapping for STH up to 2011	Perspectives for mapping of STH for 2012–2013
1	Bolivia	No	No	Mapping of STH and fascioliasis in priority areas, and for STH at national level
1	Brazil	No	A national survey for STH integrated with SCH in progress	Finalize a survey for STH and SCH at national level
1	Dominican Republic	National	No	Mapping of STH and SCH at national level
1	Ecuador	No	A national survey for STH in progress	Finalize a survey for STH at national level
1	Guatemala	No	No	Mapping of STH in priority areas
1	Guyana	Subnational integrated to LF program	No	Mapping of STH at national level
1	Haiti	National integrated to LF program	No	Mapping of STH at national level
1	Mexico	National	No	Mapping of STH at national level for evaluation of its national deworming program
1	Peru	No	No	Mapping of STH in priority areas
1	Saint Lucia	No	No	Mapping of STH and SCH at national level for evaluation of progress towards control and elimination, respectively
1	Suriname	No	Yes (no MDA needed for STH)	Mapping of STH and SCH in some priority areas within the country
2	Belize	Yes	No	Mapping of STH at national level for evaluation of its national deworming program
2	Colombia	No	No	Mapping of STH at national level
2	El Salvador	No	A national survey for STH integrated with malaria in progress	Finalize a survey of STH and malaria at national level
2	Honduras	Yes	A national survey for STH integrated with malaria in progress	Finalize a survey of STH and malaria at national level
2	Panama	No	No	Advocacy for NID at national level including STH
2	Venezuela	No	No	Mapping of STH and SCH at national level for evaluation of its epidemiological status
3	Argentina	No	No	Mapping of STH in El Chaco area
3	Paraguay	No	No	Mapping of STH at national level
3	Nicaragua	Yes	No	Mapping of STH at national level for evaluation of its national deworming program

LF: Lymphatic filariasis; SCH: Schistosomiasis; STH: Soil-transmitted helminths; NID: Neglected infectious diseases. PAHO classified countries of LAC into 4 priority groups to focalize activities for control and elimination of NID. More information can be consulted at: http://www2.paho.org/hq/dmdocuments/2010/Prioritization_NTD_PAHO_Dec_17_2010_En.pdf.

Although the methodologies recommended by PAHO/WHO to estimate STH prevalence and intensity of infection indicate that surveys among SAC are enough to establish prevalence and intensity of infection levels in a community [133], the amount of published prevalence data found in the present study for PSAC (12.0%) is very low compared with the data recovered for SAC (56.7%). The lack of information regarding PSAC is a challenge for the Region where, according to estimates made for 2011 based on the WHO-recommended algorithm [10], one fifth of PSAC and SAC were at risk of STH infections (around 13.9 and 35.4 million of a total of 61 and 155 million PSAC and SAC, respectively).

Although this indicator of children at risk of infection is an indirect way of identifying areas and population groups requiring massive deworming interventions, it does not take into account differences within countries regarding ecological zones and deworming activities that may be underway. Therefore, mapping (be it through population-based surveys, sentinel surveillance or the use of methods based on geographic information systems and remote sensing technologies that combine epidemiological, demographic, climate, social and economic variables to estimate geographic areas and populations at risk of several diseases) should be promoted in all LAC countries to identify the administrative units at second subnational level (districts, municipalities or provinces depending on the geopolitical structure of each country or territory) and focus on the implementation of deworming interventions among population groups at risk due to their living conditions. Such mapping should be promoted not only as a baseline to start deworming and other interventions in each country, but also as part of the monitoring and evaluation of progress towards STH control goals, as well as to modulate deworming activities (increasing, sustaining or reducing them).

According to data from the PAHO Regional NID Program, the countries that have conducted national mass deworming programs maintaining coverage above 75% for more than five years are Mexico (two or three rounds per year for SAC), Nicaragua (one round per year for PSAC and SAC), Dominican Republic (one round per year for SAC) and Honduras (a round per year for SAC). However, it is worth noting that despite the limitations already mentioned, our study found data published on prevalence levels above 20% and even above 50%. This may indicate that it is necessary to insist on mapping differentiated by ecological zones and population groups living in at-risk conditions that perpetuate STH transmission cycles given that national averages, and sometimes even subnational averages, can mask the real local situation.

The low amount of data published on STH intensity of infection in LAC during the period under study (151 data points from seven countries) is also noteworthy, especially the lack of data published for this indicator after 2006 at the lowest subnational administrative levels. It may be that this indicator was not estimated in the studies found, or that it was estimated but not included in the publications. However, it has been clearly documented that moderate to heavy intensity infections have significant implications for children's physical and cognitive development, as well as for severe or even fatal complications among children under 15 years of age, especially school age children [4]–[6]. Our study also found that in 63% of the data published intensity of infection ranged from moderate to heavy. It is important to suggest to health authorities in LAC countries that when massive and sustained deworming is implemented, the indicator showing the fastest change towards reduction is intensity of infection and that this has a remarkably positive impact on the well-being of children [134]. The reduction in infection prevalence will be slower, as it depends on the effect of other social determinants such as access to safe water, basic sanitation, nutrition, use of footwear and housing improvement (e.g., eliminating dirt floors, adding ventilation, lighting), amongst others.

In 2011, PAHO published an updated mapping of NIDs in LAC based on secondary sources of information [135]. The study included data on STH prevalence at first subnational level (departments, states or provinces depending on each country's characteristics) for several countries and the aim was to have an approximate idea of which areas would need to implement deworming activities at least once a year. The study also concluded that there were information gaps for several countries. Through this new search of scientific literature, PAHO's Regional NID Program wanted to analyze data availability at the lowest subnational administrative levels and learn about the status of mapping.

As indicated by WHO, mapping based on secondary sources (i.e., surveys published in recent years, official reports on prevalence surveys, undergraduate and graduate papers on STH prevalence) are useful as a first approach when the epidemiological status of a given geographic area is unknown. However, mapping quality varies according to the quality of the data published in the different secondary sources and it does not replace the power and strength of population-based studies whose aim is to establish baseline epidemiological data for STH or evaluate the impact of deworming programs, particularly those conducted at large scale and sustained through time [133].

Despite the limitations of this study, the dataset has been useful for further analysis. PAHO's Regional Neglected Infectious Diseases Program joined efforts with the Department of Epidemiology and Public Health at the Swiss Tropical and Public Health Institute to develop ecological niche-based models and Bayesian geostatistical models to predict the disease distribution at municipality level including STH. This dataset was shared to contribute to a manuscript on geostatistical meta-analysis of STH in South America whose results were published in 2013 and the data were included on the Global Database for Mapping, Control, and Surveillance of Neglected Tropical Diseases (GNTD) [136]. The dataset was shared also with the Inter-American Development Bank (IADB) as part of a joint initiative to carry out analysis on correlation between STH prevalence and some determinants as access to water and sanitation, education and housing features whose results were published in 2013 [137].

Even though by the time that this study was completed data for STH in LAC were not available on the Global Atlas for Helminth Infections (GAHI) nor on the GNTD, countries in LAC could benefit from data already available by 2013, and thus have useful data to support control activities for STH. Additionally, PAHO encourages countries in LAC to share information with the aforementioned initiatives that contribute for a better understanding of distribution of prevalence of STH and thus support countries in LAC to move forward the agenda for the control and elimination of neglected infectious diseases, in general, and in particular of STH.

### Conclusions

It is necessary and urgent to update the mapping of STH prevalence and intensity of infection in several LAC countries in order to make better evidence-based decisions regarding deworming activities. The data available for PSAC are insufficient to know the real situation of STH prevalence and intensity of infection in many countries in the LAC region, and although more data for SAC population were found, these data are only from a limited number of countries and some second administrative levels.

It is also necessary to prioritize the operational research agenda within governments and interested groups in order to develop STH mapping activities. Mapping is necessary to know where are the populations at risk and which age groups are at risk of STH infection, as well as the municipalities where authorities need to focus their efforts (if these data are available), and also for monitoring and evaluation purposes to know the impact of interventions, not only deworming, but also nutrition, education, and environmental interventions and integrated actions to reduce child morbidity and mortality and improve child development.

Without enough accurate and specific data about STH prevalence and intensity of infection by age group (PSAC and SAC) in LAC, it will be difficult to identify with certainty the main needs, resources to be assigned and places where integrated actions, including deworming, must be focused in order to reach the regional and global deworming goals for children at risk and reduce the prevalence and intensity of infections by STH.

## Supporting Information

Checklist S1 **PRISMA checklist.** *Source*: PAHO based on literature search for Latin American and the Caribbean.
(DOC)

Figure S1 **Flowchart of included studies.** *Source*: PAHO based on literature search for Latin American and the Caribbean.
(DOC)
