# Supplementary material for: Update on the Mapping of Prevalence and Intensity of Infection for Soil-Transmitted Helminth Infections in Latin America and the Caribbean: A Call for Action
Source: PLoS Negl Trop Dis. 2013 Sep 19;7(9):e2419. doi: 10.1371/journal.pntd.0002419 (PMC3777864; doi:10.1371/journal.pntd.0002419)
Supplement: Figure S1 — Flowchart of included studies. Source: PAHO based on literature search for Latin American and the Caribbean. (DOC) [file pntd.0002419.s002.doc]

**Flow Diagram**

**Screening**

**Included**

**Eligibility**

**Identification**

Records identified through database searching
(n = 534)

Additional records identified through other sources
(n = 21)

Records after duplicates removed
(n = 379)

Records screened
(n = 379)

Records excluded
(n = 143)

Full-text articles assessed for eligibility
(n =236)

Full-text articles excluded (did not met inclusion criteria
(n =116)

Studies included in qualitative synthesis
(n =120)

Studies included in quantitative synthesis (meta-analysis)
(n = NA)
